# Peony seed meal supplementation enhances semen quality in aged Qinchuan bulls

**DOI:** 10.1080/10495398.2025.2503752

**Published:** 2025-05-17

**Authors:** Shaojie Cao, Shiwei Li, Kaixue Lu, Juntai Fu, Chengwei Yang, Jiahui Qu, Juanjuan Li, Xianlin Zhao

**Affiliations:** a College of Agriculture and Bioengineering, Heze University, Heze, Shandong, China; b College of Pharmacy, Heze University, Heze, Shandong, China; c Weinan Vocational and Technical College, Weinan, Shaanxi, China

**Keywords:** Aged Qinchuan bull, peony seed meal, semen quality, semen antioxidant enzymes

## Abstract

Healthy Qinchuan bulls aged 8.5-9.5 years were selected and randomly divided into 6 groups based on 0%, 2%, 4%, 6%, 8%, and 10% dietary supplementation of peony seed meal for three months. Overall, linear motility (LM), progressive motility (PM), straight-line velocity (VSL), curvilinear velocity (VCL), and the percentage increase of all sperm grades (A to D) were affected (p<0.05) by the dietary supplementation. LM (29.5 vs. 24.1%), PM (69.4 vs. 60.0%), VLS (38.0 vs. 33.3 μm/s), and VCL (48.3 vs. 44.3 μm/s) were consistently higher in the 6% peony seed meal dietary supplementation groups than in the control group, respectively. A similar pattern was observed in the 8% group. Inversely to sperm grades C and D, grades A and B were higher in all treated groups than the control group, except for the 2% group in sperm grade A. Moreover, the levels of the enzymes Superoxide dismutase (SOD), catalase (CAT), glutathione reductase (GR; except for 2% peony seed group), and glutathione peroxidase (GSH-px) were higher than the control group. The level of malondialdehyde (MDA) was lower in all treated groups than in the control group. Dietary supplementation of 2-8% peony seed meal improves the semen quality of aged Qinchuan bulls.

## Introduction

Qinchuan cattle is a dual-purpose indigenous breed reared for draught and meat production in China. This breed is native to the Guanzhong Plain of Shaanxi Province, well adapted to harsh climatic conditions, and genetically developed for disease-resistance traits in specific ecological environments. The breed has the characteristics of combating feed scarcity and is one of the leading varieties for developing the beef cattle industry and cultivating new varieties of beef cattle.^1^ In China, the Qinchuan breed has been developed for meat quality traits through continuous rigorous research over the last three decades.^1–4^ However, with the introduction of exotic breeds and the development of agricultural mechanization, the number of Qinchuan cattle continued to decrease, and the number of excellent Qinchuan bulls showed a downward trend. The aged Qinchuan bulls often exhibit diminished semen quality, impacting their breeding value, and can threaten their genetic diversity.^5^ It is important to investigate the underlying mechanism to extend the breeding lifespan of senile Qinchuan bulls. Nutrition is important in maintaining reproductive efficiency in aged bulls.^6^

The peony seed meal is the solid residue left over from the seed of *Paeonia suffruticosa* Andr. during oil extraction.^7^ The yield of peony seed meal is 25%-30% during the preparation of peony seed oil, which is rich in polysaccharides, proteins, polyphenols, flavonoids, vitamins, and other active ingredients.^7^^,^^8^ Chemically, the peony seed meal contains by high portion of *α*-linolenic acid (>38%), a fairly low ratio of n-6 to n-3 polyunsaturated fatty acids (0.69), and a much higher content of *γ*-tocopherol than various conventional seed oils. Moreover, the peony seed meal is an excellent antioxidant as it possesses a potent scavenging effect against free radicals as well as *in vivo* antioxidant capacity by protecting living organs from oxidative damage due to various causes, such as an exogenous free radical-generating agent and lipid peroxidation from diet-induced hyperlipidemia. These antioxidant properties of the peony seed meal are closely related to the special chemical composition that is characterized by high contents of *α*-linolenic acid and *γ*-tocopherol, especially the low ratio of n-6/n-3 PUFAs originating from its uncommon abundance in *α*-linolenic acid.^7^ Every year, a large amount of peony seed meal is abandoned as oil extraction waste, causing environmental pollution and resource waste.

Flavonoids play a variety of biological activities in plants, animals, and bacteria.^9^ Peony seed meal has a variety of pharmacological and biological activities that can improve the body’s immunity and antioxidant capacity. The study of Shi Junjun^10^ showed that the polysaccharide contained in peony seed meal had potential antioxidant and anti-tumor activities. Fermented peony seed meal (FPSM) acts as an antioxidant to reduce the content of MDA in pigs’ serum.^11^ In addition, studies have shown that adding a certain amount of peony seed meal to the diet can improve the production performance of laying hens and the content of polyunsaturated fatty acids (PUFA) in egg yolk and can stimulate the immune organs of chickens to form a large number of immunoglobulins, inhibit the reproduction and growth of harmful microorganisms in the intestinal tract of animals, and enhance the body immunity of chickens.^12^^,^^13^ Previously, the effect of peony seed meal on the semen quality of aged Qinchuan cattle has not been explored. We hypothesize that a 3-month dietary supplementation of peony seed meal can improve some sperm traits of aged Qinchuan cattle. Therefore, the present study investigated the dietary impact of peony seed meal supplementation on the semen quality of aged Qinchuan cattle.

## Materials and methods

### Ethics approval

The study was approved by the ethical committee of the College of Agriculture and Bioengineering, Heze University, Heze, China, under P#ZR2019MC036.

### Study design

In total, 18 bulls of Qinchuan beef cattle aged 8.5-9.5 years old were selected with good health and the same body weight (550 ± 10kg). The bulls were clinically healthy and vaccinated against Foot-and-mouth disease twice a year (Zhongnong Weite Biotechnology Co., Ltd, China); bovine papillomatosis twice a year (Harbin Veterinary Medicine Institute, China); and brucellosis once a year (Tiankang Biotechnology Co., Ltd., China). The animals were dewormed four times a year with ivermectin (Haizheng Pharmaceutical, China) and albendazole (Changzhou Jialing Pharmaceutical Co., Ltd., China). These bulls were categorized into six groups based on the levels of peony seed meal dietary supplementation (control, 2%, 4%, 6%, 8%, and 10%) for 3 months. The chemical composition of the feed was performed at the College of Agriculture and Bioengineering, Heze University, Heze, China as dry matter (DM) 69.3%, crude protein (CP) 26.33, Ether extracts (EE) 5.2%, Ash 1.99%, crude fiber (CF) 5.23%, Ca 0.41%, and P 0.52%. The ingredient’s composition could not be mentioned here due to the privacy of the feed supplier. All animals were kept under the same feeding and management systems.

### Semen collection and processing

The semen was collected from the aged Qinchuan beef cattle with a clean and sterile artificial vagina (AV). For sterilization of the AV, it was completely dismantled, rinsed thoroughly with warm tap water (3 times), then rinsed once with demineralized water, and sterilized at least once a week, but not for all sessions. The bulls were first sexually stimulated through visual stimulation with the presence of a teaser bull and then allowed to mount. A prolonged sexual stimulation was achieved through a few false mountings (the bulls were allowed to mount but not ejaculate). During false mounts, it was carefully observed to physically deflect the sheath so that the penis does not come in contact with the hindquarters of the mounted animal. This prevents the chance of contaminating the penis or mount animal and also serves to prevent possible injury. A good ‘footing’ facility was provided for the bull and mounted bull to prevent slipping and subsequent injury. Based on initial screening, ejaculates having more than 85% morphologically normal sperm and more than 75% motile sperm were used for downstream investigation.^14^ The semen was further extended through a cryoprotective semen extender prepared as 1 gm fructose, 2.42 gm Tris, 25 mg gentamicin, 1.48 gm citric acid, 6.6 ml glycerol, 50,000 UI penicillin, 20 ml egg yolk in 0.1 L deionized water.^15^ For further processing, the semen temperature was lowered to 4 °C from 37 °C for 1.5 hours, and PVC straws (Biovet, France) were filled with 0.25 ml semen and stored at 4 °C for 2.50 hours at −120 °C. The sperm were then transferred to a liquid nitrogen tank for long-term storage at −196 °C until thawed. After 30 days, the sperm were thawed at 37.5 °C for 30 seconds and analyzed for quality parameters.

### Motility analysis of the sperms

The motility of the sperm was analyzed through the CASA setup (computer-assisted assessment of sperm motility) and pre-adjusted for bovine species (WeiLi Software, China). The straws (n = 3) were thawed initially at 37 °C for 45 seconds and then at 37 °C for 10 minutes in a water bath. The 4 µl semen sample (diluted) was placed in a 20 µm microscopic counting chamber for quality assessment (Leja, Nieuw Vennep, The Netherlands). The following semen quality parameters were analyzed: linear motility (%), progressive motility (%), straight-line velocity (VSL μm/s), average path velocity (VAP μm/s), curvilinear velocity (VCL μm/s), and the number of head-displaced sperms (HDS). Based on these parameters, sperm quality was categorized into four grades: A, B, C, and D. The sperm quality indices, such as linearity index, wobble coefficient, and mean coefficient, were measured from the above indicators and graded as A indicates progressive; B shows slow progressive movement; C category sperms are motile but without any progressive movement; D category spermatozoa are immotile.

### Analysis of the acrosomal integrity

The rate of the acrosomal integrity of the sperms was calculated through Giemsa staining microscopy with slight modifications as previously described.^16^ A thin smear of semen was prepared on a microscopic slide, air dried for 5-10 min, and fixed with formalin phosphate fixative for 15 min. After washing and drying, the slides were stained with Giemsa for 90 min, rinsed with tap water, and dried. The sperms were observed under a fluorescence microscope (LEIKA DM IRB), and the number of sperms with intact acrosomes was counted.

### Antioxidant activities assessment

To analyze sperm antioxidant activities, a semen sample of 120 µl was centrifuged for 5 minutes at a rate of 1600 × g; the supernatant was removed. A 360 µl of 1% Triton X100 was added, precipitated for 20 minutes, and centrifuged at 4000 × g for 30 minutes at RT. The supernatant, which contained a crude extract of the enzymes, was collected. The enzyme activity of the Superoxide dismutase (SOD), catalase (CAT), glutathione reductase (GR), glutathione peroxidase (GSH-px), and malondialdehyde (MDA) was measured using a commercial kit (Jiancheng Co. Ltd., China).

### Determination of sperm deformity rate

A smear was prepared from 500 µl of semen on a microscopic glass slide and stained with 50 µl of 2% eosin solution. The slide was fixed with methanol, 100 sperm were observed under 1000X of the microscope, and the sperm deformity rate was expressed as a percentage.

### Statistical analysis

The data was compiled in an MS Excel datasheet, the means were compared through one-way ANOVA using Duncan’s multiple-range test by SPSS-16 software. The p < 0.05 was considered statistically different.

## Results

**Figure 1.**
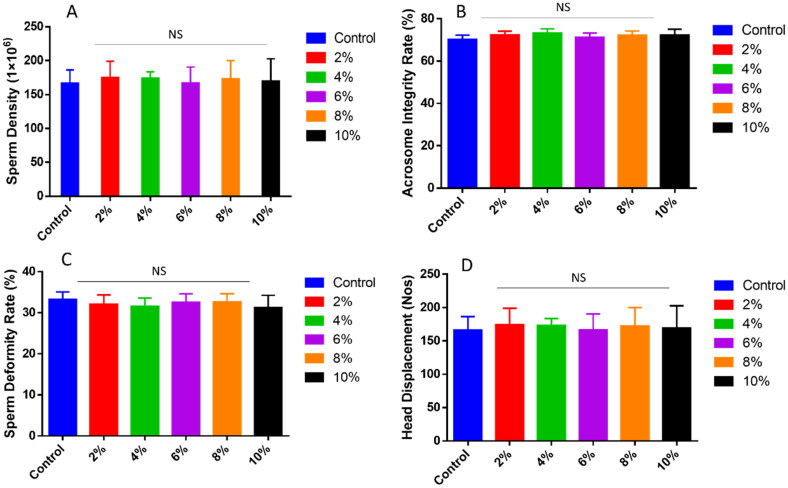
Effects of peony seed meal supplementation on sperm density, viability, acrosome integrity rate, and deformity rate of aged Qinchuan cattle.

The dietary supplementation of peony seed meal to the aged Qinchuan bulls for three months significantly (p<.05) increased sperm quality indicators as compared with the animals in the control group (Table 1). A dose-dependent improvement was observed in LM as the value increased from 24.11 ± 1.11% in the control group to a maximum of 29.45 ± 1.55% in the 6% supplementation group (p < 0.05). The LM values in all supplemented groups (2%-10%) were higher than the control, with the 4%, 6%, 8%, and 10% groups showing significant improvements. The PM followed a similar trend. While the control group recorded 60.02 ± 1.04%, the 6% and 8% supplementation groups exhibited significantly (p < 0.05) higher PM values of 69.42 ± 1.05% and 71.12 ± 1.61%, respectively. These results suggest that moderate levels of peony seed meal (6–8%) can enhance sperm motility, which is crucial for successful fertilization. The velocity parameters also showed marked improvements in response to supplementation. Straight-line velocity (VSL) increased significantly in the 4%, 6%, 8%, and 10% groups when compared with the control. The highest VSL (37.99 ± 1.53 μm/s) was recorded in the 6% group, compared to 33.33 ± 1.40 μm/s in the control. Curvilinear velocity (VCL) increased progressively from 44.27 ± 2.55 μm/s in the control group to 48.52 ± 1.52 μm/s in the 8% group. Average path velocity (VAP) also improved across all supplemented groups. Although the differences were not statistically significant (p > 0.05). Interestingly, 6-8% dietary supplementation of peony seed meal significantly (p < 0.05) improved sperm quality indicators as compared with the control group. However, no statistical variation (p > 0.05), only a numerical variation, was exhibited by sperm density, rate of acrosomal integrity, deformity rate, and number of head displacement sperms (Fig. 1).

**Table 1.** Effects of peony seed meal supplementation on sperm quality parameters in aged Qinchuan cattle.

Group	LM-%	PM-%	VSL-μm/s	VCL-μm/s	VAP-μm/s
Control	24.11 ± 1.11^a^	60.02 ± 1.04^a^	33.33 ± 1.40^a^	44.27 ± 2.55^a^	30.06 ± 4.23
2%	26.3 ± 1.21^ab^	62.06 ± 1.02^a^	36.56 ± 1.09^ab^	46.21 ± 1.70^ab^	37.66 ± 3.99
4%	27.31 ± 1.22^b^	62.11 ± 1.63^a^	37.52 ± 1.57^b^	47.13 ± 1.52^b^	38.38 ± 5.01
6%	29.45 ± 1.55^b^	69.42 ± 1.05^b^	37.99 ± 1.53^b^	48.25 ± 2.65^b^	36.43 ± 2.14
8%	27.37 ± 1.45^b^	71.12 ± 1.61^b^	36.01 ± 1.54^b^	48.52 ± 1.52^b^	39.76 ± 3.68
10%	28.32 ± 1.23^b^	62.33 ± 1.65^a^	36.02 ± 1.35^b^	47.95 ± 2.05^b^	37.23 ± 3.25

LM Linear Motility; PM Progressive motility; VSL Straight line Velocity; VCL Curvilinear Velocity; VAP Average Path Velocity. The ^a,b^different superscripts in the same column: p < 0.05.

A similar pattern was observed in the 8% peony seed meal supplementation. Inversely to sperm grades C and D, grades A and B were higher in all treated groups than the control group, except for the 2% peony seed group in sperm grade A (Table 2).

**Table 2.** Effects of peony seed meal supplementation on sperm quality of aged Qinchuan bulls.

Group	Grade A %	Grade B %	Grade C %	Grade D %
Control	16.83 ± 1.35^a^	20.22 ± 1.62^a^	52.93 ± 2.65^b^	10.03 ± 1.09^b^
2%	19.65 ± 2.22^ab^	24.86 ± 2.12^b^	47.24 ± 2.98^a^	6.86 ± 1.31^a^
4%	22.09 ± 1.98^b^	24.44 ± 2.04^b^	46.35 ± 2.51^a^	6.22 ± 1.17^a^
6%	23.12 ± 2.02^b^	24.37 ± 1.51^b^	45.13 ± 1.99^a^	6.04 ± 0.79^a^
8%	23.26 ± 2.13^b^	25.26 ± 1.94^b^	44.09 ± 2.06^a^	6.05 ± 0.97^a^
10%	22.18 ± 2.09^b^	24.15 ± 2.01^b^	46.02 ± 2.04^a^	6.11 ± 1.09^a^

The ^a,b^different superscripts in the same column: p < 0.05.

### Effect of peony seed meal on antioxidant enzyme activity in semen of aged Qinchuan cattle

The dietary supplementation of 2%-8% peony seed meal significantly (p < 0.05) increased the activities of SOD, CAT, GSH-px, and GR while decreasing the activity of MDA in the semen of aged Qinchuan breed bulls as compared to the control group (Table 3). The semen collected from the aged Qicnhuan bulls fed with 8% of dietary supplementation of peony seed meal exhibited numerically higher levels of the SOD 12.39 U/mL, the CAT 2.55 U/mL, the GSH-Px 58.22 U·mg-1 protein, and the GR 19.81 U/L than the values in the control group. The level of MDA 3.43 nmol·mg-1 protein also reduced numerically in the semen of aged Qinchuan bulls fed with 6% dietary supplementation of peony seed meal than the values in the control group (Table 3). The levels of the enzymes Superoxide dismutase (SOD), catalase (CAT), glutathione reductase (GR), and glutathione peroxidase (GSH-px) were higher than the control group (p < 0.05). The level of malondialdehyde (MDA) was lower in all treated groups than in the control group.

**Table 3.** Effects of peony seed meal supplementation on the contents of antioxidant enzyme and malondialdehyde in semen of aged Qinchuan cattle.

Group	SOD (U/mL)	CAT (U/mL) (U·mg-1 protein)	GSH-Px (U·mg-1 protein)	GR (U/L)	MDA (nmol·mg-1 protein)
Control	8.09 ± 0.12^a^	1.38 ± 0.09^a^	50.22 ± 10.23^a^	15.23 ± 1.05^a^	7.76 ± 0.15^b^
2%	11.25 ± 0.15^b^	2.33 ± 0.33^b^	57.21 ± 8.22^b^	16.95 ± 1.14^a^	4.65 ± 0.33^a^
4%	12.33 ± 0.08^b^	2.37 ± 0.28^b^	57.21 ± 8.35^b^	19.21 ± 1.12^b^	3.44 ± 0.21a
6%	11.35 ± 0.21^b^	2.49 ± 0.19^b^	58.21 ± 7.62^b^	19.22 ± 1.11^b^	3.43 ± 0.32^a^
8%	12.39 ± 0.13^b^	2.55 ± 0.25^b^	58.22 ± 9.85^b^	19.81 ± 1.09^b^	3.55 ± 0.41^a^
10%	12.38 ± 0.23^b^	2.52 ± 0.14^b^	57.21 ± 8.25^b^	19.19 ± 1.07^b^	3.57 ± 0.32^a^

The ^a,b^different superscripts in the same column: p < 0.05.

## Discussion

With the increase in animal age and the decline of sexual function, sperm quality inevitably declines. Studies have shown that sperm motility and acrosome integrity rate are closely related to the fertilization ability of sperm. These are important indicators for assessing semen quality.^17^ However, as animals enter old age, their sperm motility, viability, and various enzyme activities that contribute to the insemination process will be seriously reduced, resulting in the loss of breeding value of male animals.^18^ Therefore, in our study, the effects of the dietary supplementation of peony seed meal were assessed, and the results showed that 6-8% dietary supplementation of peony seed meal significantly increased sperm quality indicators and overall motility (p < 0.05). We only noticed that the groups 2-4% and 10% were not consistently affected by these sperm traits. Interestingly, the percentage of LM, PM, and the velocities of VSL, VCL, and VAP increased with peony seed supplementation from 6 to 8% in the diet of aged Qinchuan beef cattle than the animals in the control group. However, a further increase in the supplementation level (10%) of peony seed meal significantly (p < 0.05) reduced the PM percentage and the VCL speed. The probable reason could be its composition, as peony seed meal contains resveratrol.^19^^,^^20^ The resveratrol is a natural antioxidant and can scavenge or inhibit free radicals in semen through a high redox property of phenolic hydroxyl groups.^21^ It can effectively inhibit protein oxidation and LPO (lipid peroxidation), and preserve sperm chromatin texture, and plasma membrane integrity during oxidative stress.^22^^,^^23^ It can potentially eliminate a variety of ROS (reactive oxygen species) containing hydroxyl and Oxygen and improve sperm quality by increasing the phosphorylation of protein kinase activated by adenosine monophosphate (AMP).^24^ Moreover, in the present study, all treated groups are affected with a single exception (2%, grade A). The dietary supplementation of 8% peony seed meal significantly (p < 0.05) increased grade A and grade B sperm and reduced grade C and grade D sperms of aged Qinchuan cattle compared to the control group. The grade A and grade B sperm determine whether the sperm can effectively reach the fertilization site to a certain extent. When the percentage of grade A and grade B sperm decreases, it can interfere with sperm fertilization and reduce the fertility index. At a moderate level of 4-8% dietary supplementation, the peony seed meal improved the sperm quality parameters especially sperm motility through their active compounds such as beneficial bioactive compounds including antioxidants and fatty acids. However, at a higher level (10%) dietary supplementation causes an excessive intake of certain compounds such as saponins, flavonoids, or polyphenols may have led to oxidative stress or hormonal disruptions, negatively impacting sperm function. In the present study, the dietary supplementation of peony seed meal improves sperm motility. It may be due to the role of total flavonoids and antioxidant peptides in the main components of peony seed meal. Total flavonoids can change the fluidity of sperm membranes by dissolving phospholipids, protecting membrane lipids, and improving the permeability of sperm cells.^25–27^ It may also be that its appropriate concentration can resist the damage to the membrane, acrosome structure,^28^ and mitochondria^29^ caused by reactive oxygen species to normal sperm. Furthermore, the flavonoids maintain the structural integrity of the blood-testes barrier produced by the Sertoli cells (SCs) to protect germ cells from toxic chemicals and provide the ideal environment for spermatogenesis.^26^ In addition, the antioxidant peptides in peony seed meal can scavenge hydroxyl radicals,^30^ and maintain the body’s redox dynamic balance.^31^ The peony seed meal contains n-3 PUFAs,^7^ which contribute to the sperm cell membrane of sexually mature boars, thereby affecting the motility of flagella and improving semen motility. Furthermore, in our study, the dietary supplementation of peony seed meal significantly (p < 0.05) increased the activities of SOD, CAT, GSH-px, and GR while decreasing the activity of MDA in the semen of aged Qinchuan breed bulls as compared to the control group. Previously, that peony seed oil contains a large amount of Vitamin E and squalene, both of which improve the activity of SOD, which consequently improves the immune and antioxidant functions of the body.^19^ The peony seed meal activates the activities of many antioxidant enzymes such as catalase and superoxide dismutase through its high concentration of Resveratrol content.^32^

As a peroxide cluster ion, the ROS) is present in semen. The ROS has strong oxidizing properties, which can cause the apoptosis of sperm cells and DNA repair.^33^^,^^34^ Although sperm have the main enzyme system of defense known as reactive oxygen species (ROS), such as superoxide dismutase (SOD), catalase (CAT), and glutathione peroxidase (GSH-px), however, with the aging of animals, its concentration reduces, which results in the removal of ROS in semen,^35^ consequently leading to a serious decline in sperm quality. In the present study, the levels of the enzymes Superoxide dismutase (SOD), catalase (CAT), glutathione reductase (GR; except for 2% peony seed group) and glutathione peroxidase (GSH-px) were higher than the control group. The level of malondialdehyde (MDA) was lower in all treated groups than in the control group. It may be due to the role of flavonoids in peony seed meal, as flavonoids have strong scavenging activity on O_2_-, peroxy, and hydroxyl radicals.^36^ At the same time, studies have shown that GSH can significantly reduce H_2_O_2_-induced ROS production and protect spermatozoa; the enzymatic reaction induced by CAT enhances the body’s antioxidant capacity.^37^ In addition to that, the SOD catalyzes the conversion of O_2_- to more stable O_2_ and H_2_O_2_, reducing its harmful effect on spermatozoa. The content of SOD in semen is positively correlated with sperm motility.^38^ Because these antioxidants inhibit the production of some free radicals in the body, the damage of ROS to enzymes in semen is relatively reduced, and the concentration of malondialdehyde in semen is reduced, so the semen quality is improved.

In the present study, only 18 bulls of the Qinchuan beef cattle breed were categorized into six groups (n = 3), which limits the variation among the groups. A larger sample size would provide more robust statistical power to detect small differences, leading to exploring variation in grouping. Therefore, we found a numerically increasing tendency in the sperm quality parameters in groups from 2 to 8% dietary supplementation of peony seed meal, however, no statistical variation was found in groups. Hence, to further explore the effects of dietary supplementation of peony seed meal, the study may be performed with a larger sample size. Furthermore, another limitation of the present study is the lack of data on the chemical composition of the basal diet offered to the experimental animals. Additionally, the study lacks DNA fragmentation, in vivo fertility, and acrosome reaction capacity to explore a valid functional study of peony seed meal supplementation in aged bulls. Therefore, further research needs to be conducted to explore DNA damage through SCSA, TUNEL assays, conception rates analysis, and analysis of acrosome reaction capacity through calcium ionophore challenge.

## Conclusion

The dietary supplementation of peony seed meal was assessed. The results showed that 6-8% dietary supplementation of peony seed meal seems more consistent with increased sperm motility (p < 0.05), percentages of grade A spermatozoa, and increased activities of SOD, CAT, GSH-px, and GR while decreasing the activity of MDA in the semen of aged Qinchuan breed bulls. Therefore, the dietary supplementation of peony seed meal will extend the breeding lifespan of senile Qinchuan bulls by maintaining reproductive efficiency in aged bulls to conserve and propagate the genetic worth of elite breeding animals.

## Data Availability

The data are included in the manuscript, further details about the data will be available on request to the corresponding author.
